# High health care use prior to elective surgery for osteoarthritis is associated with poor postoperative outcomes: A Canadian population-based cohort study

**DOI:** 10.1177/13558196231213298

**Published:** 2023-12-15

**Authors:** Mayilee Canizares, J Denise Power, Anthony V Perruccio, Michael Paterson, Nizar N Mahomed, Y Raja Rampersaud

**Affiliations:** 1 Schroeder Arthritis Institute, 7989 Krembil Research Institute - University Health Network, Toronto, ON, Canada; 2 Program Lead & Interim Chief Science Officer, ICES, Toronto, ON, Canada

**Keywords:** quality-of-care indicators, high health care users, benchmarking

## Abstract

**Background:**

The characterization and influence of preoperative health care use on quality-of-care indicators (e.g., readmissions) has received limited attention in populations with musculoskeletal disorders. The purpose of this study was to characterize preoperative health care use and examine its effect on quality-of-care indicators among patients undergoing elective surgery for osteoarthritis.

**Methods:**

Data on health care use for 124,750 patients with elective surgery for osteoarthritis in Ontario, Canada, from April 1, 2015 to March 31, 2018 were linked across health administrative databases. Using total health care use one-year previous to surgery, patients were grouped from low to very high users. We used Poisson regression models to estimate rate ratios, while examining the relationship between preoperative health care use and quality-of-care indicators (e.g., extended length of stay, complications, and 90-day hospital readmissions). We controlled for covariates (age, sex, neighborhood income, rural/urban residence, comorbidities, and surgical anatomical site).

**Results:**

We found a statistically significant trend of increasing worse outcomes by health care use gradients that persisted after controlling for patient demographics and comorbidities. Findings were consistent across surgical anatomical sites. Moreover, very high users have relatively large numbers of visits to non-musculoskeletal specialists.

**Conclusions:**

Our findings highlight that information on patients’ preoperative health care use, together with other risk factors (such as comorbidities), could help decision-making when benchmarking or reimbursing hospitals caring for complex patients undergoing surgery for osteoarthritis.

## Introduction

Osteoarthritis is a highly prevalent chronic condition worldwide associated with pain, disability, and high health care use.^1^ With an aging population and increasing rates of obesity, the burden of osteoarthritis for individuals and the health care system is expected to increase in the coming years globally.^2–4^ Studies have estimated that direct health care costs will increase, primarily driven by hospitalization costs, particularly for surgeries such as joint arthroplasty.^3,4^

In an effort to curtail rising health care costs over the past decade, there has been a paradigm shift from a culture of cost containment to that of quality improvement in both Canada and the United States.^5,6^ As a result, health care systems continue to move away from global funding to value-based funding models, where funding is tied more directly to quality-of-care.^6^ It is therefore important to understand whether extraneous factors (i.e., those outside the control of surgeons or hospitals) influence quality-of-care indicators, so that resources can be efficiently allocated. Quality-of-care is often evaluated by indicators covering quality aspects on three interrelated domains: structures, processes, and health outcomes achieved by patients.^7^ Indicators of health care outcomes achieved by patients (e.g., pain, disability, and satisfaction) are seldom routinely collected, while indicators in the structure and process domains such as acute readmissions, extended length of stay (LOS), and postoperative complications are commonly used as they are easily derived from routinely collected administrative data.

Given the high volumes of elective surgeries performed for osteoarthritis, it is not surprising there have been efforts to characterize quality-of-care indicators and their drivers in this population. Studies following major elective orthopedic surgery, such as joint arthroplasty, show quality indicators are influenced by multiple factors such as age, sex, insurance status, and comorbidity.^8,9^ One additional potential factor that has received less attention is patients’ use of health services prior to surgery. The few studies that have considered it have been limited to highly selected populations (i.e., 65+ years, US military), single institutions, or specific types of surgery.^10–13^ These studies have found that, generally, preoperative high health care users had worse postoperative outcomes (e.g., readmissions) and higher costs. Nevertheless, there is still limited knowledge, from a system and health care payer perspective, on the patterns of health care use prior to surgery among the broader osteoarthritis population undergoing elective surgery.

Our study addresses this issue by (1) drawing from routinely collected population-based data on health care use to characterize health care use in the year prior to surgery for osteoarthritis and (2) examining the association of preoperative health care use with quality-of-care indicators in the structure and process domains (extended LOS, complications, hospital readmissions, emergency department [ED] visits, use of inpatient rehabilitation, and home care services) while controlling for patients’ demographic factors and comorbidity.

## Methods

### Study design and data sources

We undertook a population-based cohort study of administrative health care data from Ontario, Canada. Ontario is the country’s most populous province, accounting for almost 40% of the Canadian population. The province has a publicly funded, single payer, health care system that covers all medically necessary physician visits and hospital care. In Ontario, administrative health care use data are housed at the ICES, an independent, non-profit research institute, whose legal status under Ontario’s health information privacy law allows it to collect and analyze health care and demographic data, without consent, for health system evaluation and improvement. We used multiple administrative health care databases, linked at the patient level using unique encrypted health card numbers in a protected environment.

Inpatient hospitalizations were captured in the Canadian Institute for Health Information Discharge Abstract Database, outpatient surgery and ED visits in the National Ambulatory Care Reporting System, outpatient physician visits in the Ontario Health Insurance Plan Claims History Database, inpatient rehabilitation services in the National Rehabilitation Reporting System, and home care services in the Ontario Home Care Database. These databases included information regarding patient demographics, diagnoses recorded using International Classification for Diseases for each physician visit and hospital visit, surgical interventions coded using Canadian Classification of Intervention procedure codes, and patient and hospital identifiers.^14,15^

### Study participants

The study population was Ontarians of all ages who had elective surgery for osteoarthritis between April 1, 2015 and March 31, 2018. This produced a total of 124,750 study participants.

We used ICD-10 primary/most responsible discharge diagnosis codes M15–M19 to identify surgeries associated with osteoarthritis. These codes have been validated and used in previous studies of osteoarthritis using administrative data.^16^ We also included patients who underwent surgeries for spinal osteoarthritis, which was defined based on clinical expertise as spinal stenosis (ICD-10 code M48.0) and spondylosis (ICD-10 code M47). For patients who had multiple eligible surgeries during the accrual period, only the first surgery was included.

### Study variables

The exposure variable of interest was health care use 1 year prior to surgery. Using previously published methods,^17^ we calculated the total number of health care encounters during the year preceding surgery and grouped patients into four categories of use: very high (≥95^th^ percentile), high (90^th^–94^th^ percentile), moderate (50^th^–89^th^ percentile), and low (<50th percentile). For each study participant and in the year preceding the index surgery, we counted the total number of health care encounters, overall and by encounter type (outpatient physician visits, ED visits, day surgeries, and inpatient hospital admissions). Outpatient physician visits were further classified according to physician specialty: family or general practitioner, musculoskeletal specialists (orthopedic surgeon, neurosurgeon, and plastic surgeon), and non-musculoskeletal specialists.

Our outcome measures comprised a series of commonly used quality-of-care metrics covering the perioperative and postoperative time periods. Two binary perioperative outcomes were examined: extended LOS and the presence of complications occurring during the surgical admission. Extended LOS was defined as an LOS greater than the 75^th^ percentile LOS of the overall cohort (3 days).^8^ To identify complications occurring during the surgical admission, we used a validated algorithm based on type 2 (post-admit) ICD-10 diagnosis codes recorded on the index hospital discharge abstract (see online supplement Table S1).^18^ Postoperative binary outcomes examined were the presence of 30-day post-discharge complications, 90-day hospital readmissions, 90-day ED visits, 90-day inpatient rehabilitation admissions, and 90-day home care visits. Post-discharge complications were defined as hospital re-admissions or ED visits during the 30 days following the index hospitalization, with a primary/most responsible diagnosis among those listed in the online supplement Table S1.

Based on previously published studies,^8,9^ we included several covariates that have been shown associated with our study outcomes: age (<45, 45–64, and 65+ years), sex, neighborhood income quintile,^19^ rural versus urban/suburban residence,^20^ comorbidity (Deyo-Charlson Comorbidity Index derived from health care use during the 2 years preceding the index surgery and grouped as 0, 1, 2, 3+),^21^ surgery type (inpatient vs outpatient), and surgical anatomical site.

### Statistical analysis

We used percentages to describe the distribution of covariates and outcomes by the categories of our main exposure variable. To compare perioperative and postoperative outcomes across health care use categories while adjusting for covariates, we fit modified Poisson regression models. To compare the number of health care encounters across health care use categories, we fit linear regression models. All models were fit using generalized estimating equations to account for clustering within hospitals.^22^ We used rate ratios (RRs) with corresponding 95% confidence intervals (CIs) to report results of Poisson regression models. To test for trends in the RRs, we included a variable with the median of each health care use group in the regression models. The *p*-value associated with the type three test from the model was used to test for monotonic trends in the RRs. All analyses were performed using SAS/STAT software version 9.4.

## Results

The characteristics of the study population are presented in Table 1. Fifty-seven percent of patients were aged 65 years or older, and 71,830 (57.6%) were female. By surgical anatomical site, 74,040 (59.4%) had surgery on a knee, 35,060 (28.1%) hip, 7170 (5.7%) spine, 3600 (2.9%) elbow/shoulder, 2680 (2.1%) foot/ankle, and 2200 (1.8%) hand/wrist. The vast majority were inpatient surgeries, 109,040 (87.4%).

**Table 1.** Patient characteristics across categories of preoperative health care use among patients with surgery for osteoarthritis. Ontario, Canada, 2015–2018.

	All	Preoperative health care use			
		Low users (<50^th^ percentile)	Moderate users (50^th^–89^th^ percentile)	High users (90^th^–94^th^ percentile)	Very high users (≥95^th^ percentile)
Total	124,750 (100.0%)	55,760 (44.7%)	56,140 (45.0%)	6580 (5.3%)	6270 (5.0%)
Age group					
<45	4390 (2.6%)	2600 (4.7%)	1410 (2.5%)	180 (2.7%)	200 (3.1%)
45–64	49,450 (40.1%)	25,470 (45.7%)	19,520 (34.8%)	2110 (32.1%)	2350 (37.5%)
65+	70,910 (57.4%)	27,690 (49.7%)	35,210 (62.7%)	4290 (65.1%)	3720 (59.4%)
Sex (female)	71,830 (57.6%)	29,980 (53.8%)	34,020 (60.6%)	4060 (61.7%)	3770 (60.2%)
Neighbourhood income					
Q1 (lowest)	21,580 (17.3%)	8590 (15.4%)	10,050 (17.9%)	1260 (19.2%)	1390 (22.2%)
Q2	24,700 (19.8%)	10,760 (19.3%)	11,280 (20.1%)	1370 (20.8%)	1320 (21.0%)
Q3	25,200 (20.2%)	11,320 (20.3%)	11,400 (20.3%)	1350 (20.5%)	1200 (19.1%)
Q4	25,820 (20.7%)	12,100 (21.7%)	11,450 (20.4%)	1280 (19.4%)	1130 (17.9%)
Q5 (highest)	27,450 (22.0%)	12,990 (23.3%)	11,960 (21.3%)	1320 (20.0%)	1230 (19.6%)
Rural residence (yes)	20,440 (16.4%)	11,280 (20.2%)	7940 (14.2%)	700 (10.6%)	520 (8.2%)
Comorbidity index^a^					
0	57,290 (45.9%)	34,320 (61.6%)	20,360 (36.3%)	1460 (22.1%)	1150 (18.4%)
1	33,020 (26.5%)	13,380 (24.0%)	16,520 (29.4%)	1710 (26.0%)	1410 (22.5%)
2	18,270 (14.6%)	5510 (9.9%)	10,100 (18.0%)	1390 (21.2%)	1270 (20.2%)
3+	16,170 (13.0%)	2550 (4.6%)	9160 (16.3%)	2020 (30.7%)	2440 (38.9%)
Anatomical surgical site					
Knee	74,040 (59.4%)	32,340 (58.0%)	33,980 (60.5%)	3980 (60.5%)	3740 (59.6%)
Hip	35,060 (28.1%)	16,890 (30.3%)	15,050 (26.8%)	1650 (25.0%)	1470 (23.5%)
Spine	7170 (5.7%)	2250 (4.0%)	3690 (6.6%)	570 (8.7%)	660 (10.5%)
Elbow/shoulder	3600 (2.9%)	1650 (3.0%)	1590 (2.8%)	180 (2.7%)	180 (2.9%)
Foot/ankle	2680 (2.1%)	1530 (2.8%)	930 (1.7%)	110 (1.6%)	110 (1.7%)
Hand/wrist	2200 (1.8%)	1100 (2.0%)	900 (1.6%)	90 (1.4%)	110 (1.8%)
Surgery type					
Inpatient	109,040 (87.4%)	47,180 (84.6%)	50,300 (89.6%)	5970 (90.8%)	5590 (89.3%)
Outpatient	15,710 (12.6%)	8580 (15.4%)	5840 (10.4%)	610 (9.2%)	680 (10.7%)

^a^Deyo-Charlson Comorbidity Index is a measure of patient comorbidity prior to the index surgery and ranges from 0 (lowest complication risk) to 3 or more (highest complication risk).

Compared to low users, very high users were older (59.4% of very high users vs 49.6% of low users were 65+ years), more often female (60.2% vs 53.8%), more often lived in low-income neighborhoods (22.2% vs 15.3%), and were less likely to live in rural areas (8.2% vs 20.2%).

Very high users had a greater burden of comorbidity: 81.6% of very high users had a comorbidity index of at least one compared to 38.4% among low users, and 38.9% versus 4.6% had scores of three or more, respectively (Table 1). The frequency of recorded comorbidities is presented in online supplement Table S2.

**Figure 1.**
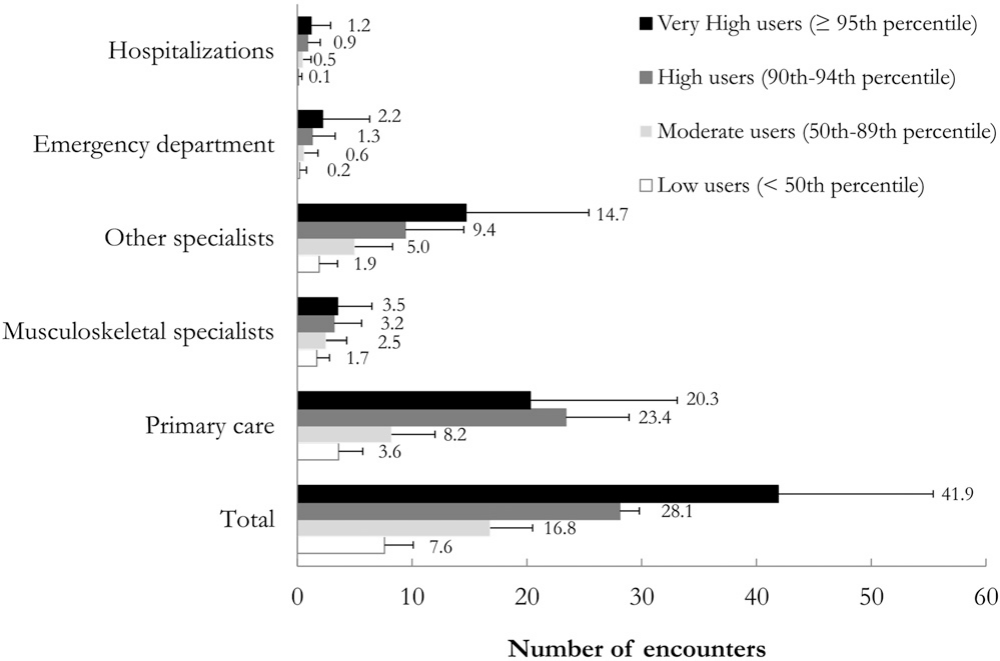
Average (and standard deviation of) number of preoperative health care encounters by encounter type and preoperative health care use category among patients with surgery for osteoarthritis (Ontario, Canada, 2015–2018).

The average ±SD number (median [IQR]) of health care encounters before surgery among very high users was 41.9 ± 13.5 (38 [34–45]) compared to 7.6 ± 2.5 (8 [6–10]) among low users (Figure 1).

There were statistically significant trends toward a higher mean number of encounters for each encounter type across gradients of health care resource use (*p* < .0001 for trend of all variables). Visits to primary care physicians consistently accounted for almost one-half of all encounters across preoperative health care use groups. Notably, visits to non-musculoskeletal specialists accounted for 25.0% of all encounters among low users and 35.1% among very high users.

In unadjusted analyses, we found strong evidence of trends of worse perioperative and postoperative outcomes according to health care use category (Table 2). For example, 45.0% of very high users had extended LOS compared to 28.3% of low users (RR, 95% CI: 1.59 (1.54, 1.64)); 7.4% of very high users had at least one complication during the surgical admission compared to 5.3% of low users (RR, 95% CI: 2.10 (1.91, 2.32)). Generally, postoperative outcomes were disproportionally worse among very high users. For instance, 8.2% of very high users had at least one complication within 30 days of discharge compared to 4.3% among low users (RR, 95% CI: 1.88 (1.72, 2.06)). Likewise, 13.9% of very high users had 90-day hospital readmissions compared to 5.9% of low-users (RR, 95% CI: 2.36 (2.20, 2.54)).

**Table 2.** Unadjusted perioperative and 90-day postoperative outcomes among patients with surgery for osteoarthritis by gradients of health care use before surgery. Ontario, Canada, 2015–2018.

	Preoperative health care use gradients							
	Low users (<50^th^ percentile)		Moderate users (50^th^–89^th^ percentile)		High users (90^th^–94^th^ percentile)		Very high users (≥95th percentile)	
	%	RR^b^	%	RR^b^ (95% CI)	%	RR^b^ (95% CI)	%	RR^b^ (95% CI)
Perioperative outcomes								
Extended length of stay^a^	28.3	1.00	38.6	1.36 (1.34, 1.39)	44.1	1.56 (1.51, 1.61)	45.0	1.59 (1.54, 1.64)
Complications (at least one)	3.5	1.00	5.3	1.51 (1.42, 1.59)	6.7	1.90 (1.72, 2.10)	7.4	2.10 (1.91, 2.32)
Postoperative outcomes								
30-day complications (at least one)	4.3	1.00	5.4	1.25 (1.19, 1.32)	6.5	1.50 (1.36, 1.66)	8.2	1.88 (1.72, 2.06)
90-day hospital readmission	5.9	1.00	8.1	1.38 (1.32, 1.44)	10.7	1.82 (1.68, 1.96)	13.9	2.36 (2.20, 2.54)
90-day emergency department visit	16.0	1.00	20.3	1.27 (1.24, 1.30)	26.3	1.65 (1.58, 1.72)	31.1	1.95 (1.87, 2.03)
90-day inpatient rehabilitation admission	7.8	1.00	14.1	1.80 (1.74, 1.86)	18.9	2.41 (2.28, 2.56)	22.1	2.82 (2.67, 2.98)
90-day home care visit	50.3	1.00	55.5	1.10 (1.09, 1.12)	59.5	1.18 (1.16, 1.21)	59.5	1.18 (1.16, 1.21)

RR: rate ratio; CI: confidence interval.

^a^Defined as >75^th^ percentile.

^b^Rate ratios were obtained from regression model with log link function and Poisson distribution, using Generalized Estimating Equations to account for clustering within hospitals.

These patterns, although attenuated, persisted after controlling for age, sex, neighborhood income quintile, rural/urban residence, comorbidity index, and surgical anatomical site for all outcomes with the exception of home care visits, where differences were no longer statistically significant (Table 3). Full models are presented in online supplement Tables S3a & S3b. Furthermore, we found similar patterns when analyses were stratified by surgical anatomical site (online supplement Table S4).

**Table 3.** Adjusted^a^ rate ratios for the association between perioperative and postoperative outcomes with gradients of health care use within the year prior to surgery among patients with surgery for osteoarthritis. Ontario, Canada, 2015–2018.

	Preoperative health care use gradients (reference: Low users [<50th percentile users])					
	Moderate users (50^th^–89^th^ percentile)		High users (90^th^–94^th^ percentile)		Very high users (≥95^th^ percentile)	
	aRR	95% CI	aRR	95% CI	aRR	95% CI
Perioperative						
Extended length of stay^b^	1.14	(1.12, 1.16)	1.20	(1.17, 1.24)	1.23	(1.19, 1.27)
Complications (at least one)	1.23	(1.16, 1.30)	1.40	(1.26, 1.56)	1.55	(1.40, 1.73)
Postoperative						
30-day complications (at least one)	1.23	(1.17, 1.30)	1.46	(1.31, 1.62)	1.84	(1.66, 2.02)
90-day hospital readmission	1.26	(1.20, 1.32)	1.54	(1.42, 1.67)	1.92	(1.78, 2.08)
90-day emergency department visit	1.24	(1.20, 1.27)	1.56	(1.49, 1.64)	1.81	(1.73, 1.90)
90-day inpatient rehabilitation admission	1.32	(1.27, 1.37)	1.56	(1.47, 1.65)	1.82	(1.72, 1.93)
90-day home care visit	0.98	(0.97, 0.99)	1.00	(0.98, 1.02)	1.01	(0.99, 1.03)

*Note:* Rate ratios obtained from regression model with log link function and Poisson distribution using Generalized Estimating Equations to account for clustering within hospitals.

aRR: adjusted rate ratio; CI: confidence interval.

^a^Models adjusted for age, sex, neighborhood income quintiles, rural/urban residence, Charlson Comorbidity Index, surgical anatomical site, and type of surgery.

^b^Extended length of stay, defined as >75^th^ percentile.

## Discussion

We studied a wide range of health care services used by patients undergoing surgery for osteoarthritis in a publicly funded and single payer health care system. We found that compared to low preoperative health care users, very high users were more likely to have extended LOS, experience complications (perioperative and 30-day post-discharge), have higher rates of hospital readmissions, ED visits, and use inpatient rehabilitation services more frequently within 90 days following surgery. These findings persisted even after adjusting for the higher comorbidity burden among very high users. Similar patterns were observed across surgical anatomical sites.

The few studies that have examined the impact of preoperative health care use on postoperative outcomes have been limited to specific populations, single institutions, or specific types of surgery.^10–13^ Our study expands on these works as we comprehensively examined the influence of preoperative health care use on commonly used quality-of-care metrics among patients of all ages undergoing major elective surgical procedures for osteoarthritis. Consistent with the abovementioned studies, we found that, compared to low users, very high users were more likely to have worse perioperative and postoperative outcomes (with the exception of home care visits) independently of their comorbidities and demographic characteristics. Importantly, these associations were consistent across the broader osteoarthritis surgical population, regardless of surgical anatomical site. Furthermore, the independent effects of preoperative health care use on outcomes suggest that preoperative service use may reflect persistent behavior or serve as a marker of patient complexity beyond the factors that are routinely considered.

The higher burden of comorbidity among very high health care users is in line with previous studies on the general population.^23,24^ Our specific finding of high number of non-musculoskeletal specialist visits among very high users is likely explained by the high burden of comorbidity in this sub-population – 81.6% of very high users had comorbidities. Studies have suggested that patients benefit from attention to their comorbidities prior to surgery, as the risks of complications and poor outcomes associated with surgery are compounded by underlying comorbidity.^25,26^

Preoperative health care use is a holistic measure that could be considered along with comorbidity when evaluating the complexity of patients undergoing elective surgery for osteoarthritis. This is particularly relevant in light of the health system funding reforms that have taken place in Ontario and elsewhere over the past decade.^6,27,28^ There has been a move away from a global funding system to value-based models in which hospitals are compensated based on the number of patients they treat, the services they deliver, and the quality of those services. Among these initiatives, bundled payment models have been implemented in pilot sites across Ontario and in 2018, more than 60% of hip and knee arthroplasties were planned to be funded using bundle payments.^27^ In bundled care models, health care providers receive a single payment per patient to cover all their care needs for specific health problems. These models are designed to counter fragmented care delivery and rising system costs by bundling services and encouraging care coordination and collaboration. Under such paradigms, it is important to anticipate the postoperative care needs of patients in order to appropriately allocate funds. Patients’ age and comorbidities are usually key factors used for risk-adjustments of quality indicators. Our findings show that, over and above comorbidity, health care use prior to surgery provides important additional information to the estimation of bundled payments. Therefore, preoperative health care use could be used in conjunction with other risk factors when benchmarking or reimbursing hospitals caring for complex patients undergoing surgery for osteoarthritis.

## Limitations

Our study has five main limitations. First, although more than 90% of Ontario physicians are paid on a fee-for-service basis or shadow bill,^29^ care delivered by salaried physicians and those working under other alternative payment plans was not included in this study. The lack of detailed clinical information in administrative data limits our ability to adjust for disease severity in our models and may also have limited our ability to appropriately identify perioperative complications. Nevertheless, we identified perioperative complications using ICD-10 diagnosis codes that have been validated for use in administrative data.^18^ We created our study cohort using ICD-10 codes for the most responsible/principal diagnosis associated with the surgery. The codes selected to identify osteoarthritis, with the exception of spinal osteoarthritis, have been previously validated.^16^ To the best of our knowledge, there are no validated case definitions for spinal osteoarthritis (5.7% of our sample). Our use of codes for spinal stenosis (80.1% of all spine cases) and spondylosis (19.9% of all spine cases) was selected based on clinical expertise but requires further validation.

Second, we lacked important information regarding patient-reported outcomes, such as pain and disability. Future research is needed to investigate how preoperative health care use relates to health outcomes that are important to patients. Even though universal coverage in Ontario has eliminated many obstacles to accessing health care, barriers remain.^30^ It is well documented that remote rural areas have poorer access to health services than major urban centers. We found lower proportions of rural residents in the higher use categories than low users. This may be partially related to difficulties accessing health services in rural areas.

Third, although our analyses incorporated the Deyo-Charlson Comorbidity Index, which includes a long list of comorbidities, we acknowledge the potential for residual confounding by excluded comorbidities or other factors we did not – or could not – capture with administrative health data.

Fourth, we focused on elective surgery for osteoarthritis in a universal-funded health care setting. It is unclear whether our findings might generalize to other procedures and conditions, or to other jurisdictions.

Fifth, we studied an equally weighted composite exposure variable. It is possible that weights for individual components may vary depending on the outcome under consideration. Another potential aspect requiring further examination is the selection of the preoperative time frame. In this paper, we used a 1-year window and in sensitivity analysis we used a 90-day window showing similar results. Nevertheless, further methodological work addressing these issues is warranted.

## Conclusions

Our study found that osteoarthritis patients that were high users of health care in the year before surgery had poorer perioperative and postoperative outcomes. These included more frequent complications, longer LOS, and more frequent hospital and ED admissions. Future work to clarify the factors underlying these associations is important so that preoperative interventions for high users can be further optimized to improve outcomes.

Furthermore, we found that very high users have large numbers of visits to non-musculoskeletal specialists. This potentially is a reflection of their diverse health care needs that are beyond the reasons for undergoing surgery for osteoarthritis. Therefore, it is important to identify gaps in current care and focus efforts in delivering care to tailoring services according to their needs.

Our findings also reinforce the importance of appropriately accounting for these patients when reimbursing hospitals caring for high-need patients under alternative payment paradigms, such as bundled payments.

## Supplemental Material

Supplemental Material - High health care use prior to elective surgery for osteoarthritis is associated with poor postoperative outcomes: A Canadian population-based cohort study Supplemental Material for High health care use prior to elective surgery for osteoarthritis is associated with poor postoperative outcomes: A Canadian population-based cohort study by Mayilee Canizares, J Denise Power, Anthony V Perruccio, Michael Paterson, Nizar N Mahomed, and Y Raja Rampersaud in Journal of Health Services Research & Policy

## References

[1] Cui A Li H Wang D , et al. Global, regional prevalence, incidence and risk factors of knee osteoarthritis in population-based studies. EClinicalMedicine 2020; 29-30: 100587. 34505846 10.1016/j.eclinm.2020.100587 PMC7704420

[2] Ackerman IN Bohensky MA Zomer E , et al. The projected burden of primary total knee and hip replacement for osteoarthritis in Australia to the year 2030. BMC Muscoskel Disord 2019; 20: 90. 10.1186/s12891-019-2411-9 PMC6387488 30797228

[3] Sharif B Kopec J Bansback N , et al. Projecting the direct cost burden of osteoarthritis in Canada using a microsimulation model. Osteoarthritis Cartilage 2015; 23: 1654–1663. 26050868 10.1016/j.joca.2015.05.029

[4] Leifer VP Katz JN Losina E . The burden of OA-health services and economics. Osteoarthritis Cartilage 2022; 30: 10–16. 34023527 10.1016/j.joca.2021.05.007 PMC8605034

[5] Beaule PE Roffey DM Poitras S . Continuous quality improvement in orthopedic surgery: changes and implications with health system funding reform. Can J Surg 2016; 59: 149–150. 27240282 10.1503/cjs.005416 PMC4982857

[6] Porter ME . A strategy for health care reform--toward a value-based system. N Engl J Med 2009; 361: 109–112. 19494209 10.1056/NEJMp0904131

[7] Donabedian A . The quality of care. How can it be assessed? JAMA 1988; 260: 1743–1748. 3045356 10.1001/jama.260.12.1743

[8] Kobayashi K Ando K Kato F , et al. Predictors of prolonged length of stay after lumbar interbody fusion: a multicenter study. Global Spine J 2019; 9: 466–472. 31431867 10.1177/2192568218800054 PMC6686383

[9] Swiggett SJ Mannino A Vakharia RM , et al. Impact of biological sex on complications, lengths of stay, readmission rates, and costs of care following primary total knee arthroplasty. J Knee Surg 2021. 2021;35:1306–1311. 33545731 10.1055/s-0041-1723014

[10] Creager AE Kleven AD Kesimoglu ZN , et al. The impact of pre-operative healthcare utilization on complications, readmissions, and post-operative healthcare utilization following total joint arthroplasty. J Arthroplasty 2022; 37: 414–418. 34793857 10.1016/j.arth.2021.11.018 PMC8857028

[11] Clewley D Rhon D Flynn T , et al. Health seeking behavior as a predictor of healthcare utilization in a population of patients with spinal pain. PLoS One 2018; 13: e0201348. 30067844 10.1371/journal.pone.0201348 PMC6070259

[12] Clewley D Rhon DI Flynn TW , et al. Does health care utilization before hip arthroscopy predict health care utilization after surgery in the US Military Health System? An investigation into health-seeking behavior. J Orthop Sports Phys Ther 2018; 48: 878–886. 30032699 10.2519/jospt.2018.8259

[13] Hyer JM Tsilimigras DI Paredes AZ , et al. Is annual preoperative utilization an indicator of postoperative surgical outcomes? A study in medicare expenditure. World J Surg 2020; 44: 108–114. 31531723 10.1007/s00268-019-05184-8

[14] Canadian Institute for Health Information . Canadian coding standards for version 2018 ICD-10-CA and CCI, https://secure.cihi.ca/free_products/CodingStandards_v2018_EN.pdf, (2018, accessed 15 February 2021).

[15] Ontario Ministry oh Health & Ministry of Long-term Care . Schedule of benefits for physician services under the health insurance act, http://www.health.gov.on.ca/english/providers/program/ohip/sob/physserv/physserv_mn.html (accessed 15 February 2021).

[16] Lix LM Yogendran MS Shaw SY , et al. Population-based data sources for chronic disease surveillance. Chron Dis Can 2008; 29: 31–38. 19036221

[17] Wodchis WP Austin PC Henry DA . A 3-year study of high-cost users of health care. Can Med Assoc J 2016; 188: 182–188. 26755672 10.1503/cmaj.150064 PMC4754179

[18] Southern DA Burnand B Droesler SE , et al. Deriving ICD-10 codes for patient safety indicators for large-scale surveillance using administrative hospital data. Med Care 2017; 55: 252–260. 27635599 10.1097/MLR.0000000000000649

[19] Canadian Institute for Health Information . Trends in income-related health inequalities in Canada, methodology notes, https://www.cihi.ca/sites/default/files/cphi-etool-meth-notes_en.pdf (accessed 15 February 2021).

[20] Kralj B. Measuring rurality – RIO2008_BASIC: methodology and results, https://www.oma.org/wpcontent/uploads/2008rio-fulltechnicalpaper.pdf (2008, accessed 3 November 2023).

[21] Deyo RA Cherkin DC Ciol MA . Adapting a clinical comorbidity index for use with ICD-9-CM administrative databases. J Clin Epidemiol 1992; 45: 613–619. 1607900 10.1016/0895-4356(92)90133-8

[22] McNutt L-A Wu C Xue X , et al. Estimating the relative risk in cohort studies and clinical trials of common outcomes. Am J Epidemiol 2003; 157: 940–943. 12746247 10.1093/aje/kwg074

[23] Andriotti T Dalton MK Jarman MP , et al. Super-Utilization of the emergency department in a universally insured population. Mil Med 2021; 186: e819–e825. 33247301 10.1093/milmed/usaa399

[24] Shubeck SP Thumma JR Dimick JB , et al. Hot spotting as a strategy to identify high-cost surgical populations. Ann Surg 2019; 269: 453–458. 29342019 10.1097/SLA.0000000000002663

[25] Harari D Hopper A Dhesi J , et al. Proactive care of older people undergoing surgery ('POPS'): designing, embedding, evaluating and funding a comprehensive geriatric assessment service for older elective surgical patients. Age Ageing 2007; 36: 190–196. 17259638 10.1093/ageing/afl163

[26] Leeds IL Canner JK Gani F , et al. Increased healthcare utilization for medical comorbidities prior to surgery improves postoperative outcomes. Ann Surg 2020; 271: 114–121. 29864092 10.1097/SLA.0000000000002851 PMC8559326

[27] Jacobs J Daniel I Baker GR , et al. Bundling care and payment: evidence from early-adopters, 2015, http://ihpme.utoronto.ca/wpcontent/uploads/2016/03/Walter-W2434_OHA_Bundling_Care_Payment_Policy_paper.pdf (accessed 15 February 2021).

[28] O'Reilly J Busse R Häkkinen U , et al. Paying for hospital care: the experience with implementing activity-based funding in five European countries. Health Econ Pol Law 2012; 7: 73–101. 10.1017/S1744133111000314 22221929

[29] Schultz SGR Graves E Schull M , et al. Payments to Ontario physicians from ministry of health and long-term care sources: update 2005/06 to 2017/18. Toronto, ON: Institute for Clinical Evaluative Sciences, 2019.

[30] Sibley LM Weiner JP . An evaluation of access to health care services along the rural-urban continuum in Canada. BMC Health Serv Res 2011; 11: 1–11. 21281470 10.1186/1472-6963-11-20 PMC3045284

