# Supplementary material for: High health care use prior to elective surgery for osteoarthritis is associated with poor postoperative outcomes: A Canadian population-based cohort study
Source: J Health Serv Res Policy. 2023 Dec 15;29(2):92–9. doi: 10.1177/13558196231213298 (PMC10910823; doi:10.1177/13558196231213298)
Supplement: Supplemental Material - High health care use prior to elective surgery for osteoarthritis is associated with poor postoperative outcomes: A Canadian population-based cohort study [file sj-pdf-1-hsr-10.1177_13558196231213298.pdf]

Journal of Health Services Research & Policy
High healthcare use prior to elective surgery for osteoarthritis is associated with poor post-operative outcomes: A population-based cohort study
Canizares M et al

Online Supplement

Table of Contents

Definition of adverse events 2

Table S1. ICD-10 codes to identify complications. Adapted from Southern et. al. [1] 2

Table S2. Distribution of comorbidities by gradients of pre-operative healthcare use among patients with surgery for osteoarthritis. Ontario, Canada, 2015/16-2017/18. 4

Table S3a. Adjusted models for peri-operative and 90-day post-operative outcomes among patients undergoing surgery for osteoarthritis by gradients of pre-operative healthcare use and surgical anatomical site. Ontario, Canada, 2015/16-2017/18. 5

Table S3b. Adjusted models for peri-operative and 90-day post-operative outcomes among patients undergoing surgery for osteoarthritis by gradients of pre-operative healthcare use and surgical anatomical site. Ontario, Canada, 2015/16-2017/18. 6

Table S4. Peri-operative and 90-day post-operative outcomes among patients undergoing surgery for osteoarthritis by gradients of pre-operative healthcare use and surgical anatomical site. Ontario, Canada, 2015/16-2017/18. 7

Definition of adverse events

DAD/NACRS databases collect diagnosis codes associated with the hospital admission. Since 2002, these diagnoses are coded using the International Classification of Diseases 10th Revision (ICD10-CA). Complications were identified by diagnosis codes as listed in Table S1. The following measures were calculated:

Complications: using diagnosis codes restricted to diagnosis type=2 (post-admit comorbidity) from the hospital admission as listed in Table 1.

30-day post-discharge complications: if patient had hospital re-admissions or Emergency Department visits 30 days after discharge where the main diagnosis code is as listed in Table S1.

Table S1. ICD-10 codes to identify complications. Adapted from Southern et. al. [1]

Category	ICD-10 code
Hospital-acquired infection	A020, A021, A044, A045, A047, A048, A049, A080, A081, A410, A411, A412, A414, A4150, A4151, A4152, A4158, A4180, A4188, A419, A490, B309, B373, B374, B377, B3780, B3781, B956, B957, B958, B961, B962, B964, B965, B9681, B9688, B974, G003, J150, J151, J152, J155, J156, J159, J181, J210, J853, J860, J869, J9501, K650, N390, N9951, O7530, O8500, O8600, O8610, O8620, O8630, O8680, P360, P361, P362, P363, P364, P368, P369, P38, R572, T814, T826, T827, T835, T836, T8453, T8454, T8460, T8461, T8463, T8464, T8465, T8468, T847, T857, T8742, T8746, T8747, T8748
Decubitus Ulcer	L890, L891, L892, L893, L898, L899
Endocrine & Metabolic Complications (electrolyte abnormalities, diabetes, etc.)	E1010, E1063, E1064, E110, E1110, E1111, E1163, E1164, E1363, E1463, E15, E160, E272, E891, E892, E893, G372, T503
Venous Thromboembolic Events	I260, I269, I801, I802, I822, O8710
Cardiac Complications	I200, I201, I2088, I209, I210, I211, I212, I213, I214, I219, I220, I221, I228, I229, I461, I469, I472, I481, I4900, I4901, I500, I501, I509, J81, O7420, S26811, T820, T821, T822, T825, T826, T827, T828, T829
Respiratory Complications	J150, J151, J152, J155, J156, J159, J181, J210, J3801, J3802, J3809, J690, J698, J853, J860, J869, J942, J9500, J9501, J9502, J9503, J9508, J951, J952, J955, J9580, J9581, J9588, J959, J960, S202, S22200, S22300, S22400, S22410, S22490, S27000, S27001, S27100, S27200, S27300, S27310, T173, T174, T175, T178, T179, T71, T797, T8181
Hemorrhagic Events	D62, D683, J942, J9500, O7170, O7180, O7200, O7210, O7220, O9020, P120, S064, S065, S066, S27100, S27200, S27300, S36090, S36091, S36150, S36151, S36800, S36810, S37000, S37300, T792, T810
Drug Related Adverse Events	D683, E160, E883, H910, I952, O7450, T360, T361, T365, T368, T369, T378, T380, T383, T390, T391, T393, T398, T402, T403, T404, T406, T412, T413, T420, T421, T424, T426, T427, T430, T432, T434, T435, T438, T445, T447, T450, T451, T455, T457, T458, T460, T461, T462, T464, T465, T474, T480, T486, T490, T501, T502, T509, T808, T809, T8180, T882, T883, T886
Adverse events related to fluid management	E860, E868, E877, G372, T503, T808, T809
Complications Directly Related to Surgery	H5980, M966, O7540, O8600, O9000, S26811, S27001, S36091, S36151, S36411, S36461, S37111, S37211, S37311, T810, T811, T812, T813, T8152, T8158, T8159, T816, T8181, T8188, T819
Traumatic injuries (non-procedural) arising in	S0100, S0101, S0110, S0120, S0130, S0140, S0150, S0170, S0180, S0190, S02000, S02100, S02200, S02300, S02480, S02490, S025, S02890, S030, S050, S051, S058,

hospital	S059, S060, S061, S0625, S0635, S064, S065, S066, S0685, S069, S090, S098, S099, S101, S109, S1348, S1438, S202, S204, S208, S22200, S22300, S22400, S22410, S22490, S27000, S27100, S27200, S27300, S27310, S27810, S27860, S300, S301, S3080, S3081, S3088, S309, S31200, S31400, S32100, S32400, S32500, S32700, S32800, S335, S351, S352, S355, S36090, S36150, S36460, S36610, S36810, S37000, S37090, S37110, S37190, S37210, S37290, S37300, S37310, S37390, S37610, S3908, S398, S399, S400, S408, S409, S4110, S4111, S42010, S42020, S42090, S42190, S42200, S42210, S42220, S42280, S42290, S42300, S42390, S42400, S42480, S43000, S43090, S43100, S4600, S4608, S497, S498, S499, S500, S501, S507, S508, S509, S5100, S5101, S5170, S5180, S5190, S52000, S52100, S52300, S52500, S52580, S52590, S52600, S52800, S598, S599, S600, S602, S607, S608, S609, S6100, S6170, S6180, S6190, S62000, S62500, S62690, S62800, S63100, S6359, S698, S699, S700, S701, S708, S709, S7110, S7111, S72000, S72010, S72080, S72090, S72100, S72190, S72200, S72300, S72410, S72420, S72490, S72800, S72900, S73000, S73090, S7418, S750, S799, S800, S801, S807, S808, S809, S8100, S8101, S8180, S8181, S8190, S8191, S82000, S82100, S82200, S82300, S82400, S82500, S82600, S82800, S82890, S836, S898, S899, S900, S901, S903, S907, S908, S909, S9100, S9110, S9120, S9130, S92000, S92300, S92400, S92500, S9349, S998, S999, T001, T008, T009, T090, T110, T111, T130, T131, T140, T149, T200, T202, T210, T211, T212, T213, T220, T224, T230, T232, T240, T242, T250, T252, T71, T792, T796, T797
Anesthesia related complications	O2950, O7420, O7430, O7450, O7460, O7480, O8940, O8950, O8980, T412, T413, T882, T883, T884, T885
Delirium	F050, F051, F058, F059
Central Nervous System Complications	E110, E15, F050, F051, F058, F059, G003, G372, G972, O7430, O8940, S060, S061, S0625, S0635, S064, S065, S066, S0685, S069
Gastrointestinal	A020, A044, A045, A047, A048, A049, A080, A081, B3780, B3781, K223, K650, K910, K913, S27810, S27860, S36150, S36151, S36411, S36460, S36461, S36610, T181, T182, T183, T189, T282, T855
Severe life or major vital organ threatening adverse event	G372, I210, I211, I212, I213, I214, I219, I220, I221, I228, I229, I260, I461, I469, I472, I4900, I4901, J960, K223, K650, O7420, O7420, O7430, O7510, O7540, R571, R572, R578, T71, T800, T805, T811, T882, T883, T884, T886

1. Southern DA, Burnand B, Droesler SE, Flemons W, Forster AJ, Gurevich Y, et al. Deriving ICD-10 Codes for Patient Safety Indicators for Large-scale Surveillance Using Administrative Hospital Data. Medical care. 2017;55(3):252-60.

Table S2. Distribution of comorbidities by gradients of pre-operative healthcare use among patients with surgery for osteoarthritis. Ontario, Canada, 2015/16-2017/18.

		Pre-operative healthcare use gradients			
	**All**	**Low users** (<50th percentile)	**Moderate users** (50th – 89th percentile)	**High users** (90th – 94th percentile)	**Very high users** (≥95th percentile)
	n (%)	n (%)	n (%)	n (%)	n (%)
Diabetes	27246 (21.7)	7896 (14.1)	14525 (25.8)	2255 (34.2)	2570 (40.9)
Cancer	17184 (13.7)	4568 (8.1)	9388 (16.7)	1577 (23.9)	1651 (26.3)
Chronic pulmonary disease	15066 (12.0)	4076 (7.3)	7883 (14.0)	1458 (22.1)	1649 (26.3)
Myocardial infarction	14398 (11.5)	3614 (6.4)	7919 (14.1)	1329 (20.2)	1536 (24.5)
Connective tissue	9351 (7.5)	2622 (4.7)	5036 (8.9)	819 (12.4)	874 (13.9)
Peripheral vascular disease	5066 (4.0)	1110 (2.0)	2743 (4.9)	583 (8.8)	630 (10.0)
Cerebrovascular disease	3667 (2.9)	790 (1.4)	1977 (3.5)	407 (6.2)	493 (7.9)
Congestive heart failure	3579 (2.9)	583 (1.0)	1975 (3.5)	450 (6.8)	571 (9.1)
Renal disease	3385 (2.7)	600 (1.1)	1862 (3.3)	377 (5.7)	546 (8.7)
Mild liver disease	2214 (1.8)	499 (0.9)	1160 (2.1)	249 (3.8)	306 (4.9)
Dementia	2161 (1.7)	448 (0.8)	1149 (2.0)	253 (3.8)	311 (5.0)
Peptic ulcer	1825 (1.5)	349 (0.6)	1006 (1.8)	189 (2.9)	281 (4.5)

Table S3a. Adjusted models for peri-operative and 90-day post-operative outcomes among patients undergoing surgery for osteoarthritis by gradients of pre-operative healthcare use and surgical anatomical site. Ontario, Canada, 2015/16-2017/18.

	**Rate Ratio (95% confidence Interval)**		
	**Extended LOS[§]**	**Complications**	**30-day complications**
**Healthcare use 1year before surgery** (Ref: Low users)			
Very high users (≥95th percentile)	1.23 (1.19, 1.27)	1.55 (1.40, 1.73)	1.84 (1.66, 2.02)
High users (90th-94th percentile)	1.20 (1.17, 1.24)	1.40 (1.26, 1.56)	1.46 (1.31, 1.62)
Moderate users (50th-89th percentile)	1.14 (1.12, 1.16)	1.23 (1.16, 1.30)	1.23 (1.17, 1.30)
**Age** (5 years increments)	1.11 (1.09, 1.12)	1.20 (1.19, 1.22)	1.04 (1.03, 1.06)
**Sex** (Female vs. male)	1.18 (1.16, 1.20)	1.09 (1.04, 1.15)	0.90 (0.85, 0.94)
**Income** (Ref: Lowest quintile)			
Q2	0.95 (0.93, 0.97)	0.90 (0.83, 0.98)	0.96 (0.89, 1.03)
Q3	0.93 (0.90, 0.95)	0.90 (0.83, 0.97)	0.88 (0.82, 0.95)
Q4	0.91 (0.89, 0.93)	0.91 (0.84, 0.98)	0.88 (0.82, 0.95)
Q5 (Highest)	0.91 (0.89, 0.93)	0.87 (0.80, 0.94)	0.84 (0.77, 0.90)
**Residence** (Rural vs. Urban)	0.92 (0.90, 0.94)	1.01 (0.94, 1.08)	1.42 (1.34, 1.51)
**Comorbidities** (Ref: 0)			
1	1.13 (1.11, 1.15)	1.17 (1.09, 1.25)	1.13 (1.07, 1.20)
2	1.09 (1.06, 1.11)	1.16 (1.07, 1.25)	1.18 (1.10, 1.27)
3+	1.16 (1.14, 1.19)	1.43 (1.33, 1.55)	1.16 (1.07, 1.25)
**Anatomical surgical site** (Ref: Knee)			
Hip	1.25 (1.23, 1.27)	1.49 (1.41, 1.57)	1.00 (0.95, 1.06)
Spine	1.23 (1.20, 1.27)	2.62 (2.42, 2.83)	0.95 (0.86, 1.05)
Elbow/Shoulder			0.62 (0.52, 0.74)
Hand/Wrist	0.21 (0.19, 0.24)	0.68 (0.55, 0.84)	0.73 (0.59, 0.90)
Foot/Ankle			0.60 (0.48, 0.75)
**Extended LOS[§]** (Yes vs. No)	-	-	0.85 (0.80, 0.90)
**Adverse events** (1+ vs. 0)	-	-	1.32 (1.19, 1.46)

[§] LOS= length of stay. Extended LOS defined as ≥ 75th percentile. Rate ratios obtained from regression model with log link function and Poisson distribution using Generalized Estimating Equations to account for clustering within hospitals. Surgical sites were grouped as knee, hip spine and other (Elbow/shoulder, hand/wrist, foot/ankle) in the models for extended LOS, adverse events and inpatient rehabilitation to accommodate smaller sample sizes.

Table S3b. Adjusted models for peri-operative and 90-day post-operative outcomes among patients undergoing surgery for osteoarthritis by gradients of pre-operative healthcare use and surgical anatomical site. Ontario, Canada, 2015/16-2017/18.

	**Rate Ratio (95% confidence Interval)**			
	**90-day Hospital readmissions**	**90-day Emergency department visits**	**90-day Inpatient rehabilitation**	**90-day Home care**
**Healthcare use 1year before surgery** (Ref: Low users)				
Very high users (≥95th percentile)	1.92 (1.78, 2.08)	1.81 (1.73, 1.90)	1.82 (1.72, 1.93)	1.01 (0.99, 1.03)
High users (90th-94th percentile)	1.54 (1.42, 1.67)	1.56 (1.49, 1.64)	1.56 (1.47, 1.65)	1.00 (0.98, 1.02)
Moderate users (50th-89th percentile)	1.26 (1.20, 1.32)	1.24 (1.20, 1.27)	1.32 (1.27, 1.37)	0.98 (0.97, 0.99)
**Age** (5 years increments)	1.05 (1.04, 1.06)	1.03 (1.02, 1.04)	1.20 (1.19, 1.21)	1.07 (1.06, 1.08)
**Sex** (Female vs. male)	0.78 (0.75, 0.81)	0.95 (0.93, 0.97)	1.41 (1.36, 1.46)	1.07 (1.05, 1.09)
**Income** (Ref: Lowest quintile)				
Q2	1.00 (0.94, 1.07)	0.95 (0.91, 0.98)	0.94 (0.90, 0.99)	0.96 (0.95, 0.98)
Q3	0.95 (0.89, 1.020	0.89 (0.86, 0.93)	0.90 (0.86, 0.94)	0.94 (0.92, 0.95)
Q4	0.99 (0.93, 1.050	0.87 (0.84, 0.90)	0.95 (0.91, 0.99)	0.92 (0.91, 0.94)
Q5 (Highest)	0.94 (0.88, 1.00)	0.83 (0.80, 0.86)	0.90 (0.86, 0.94)	0.87 (0.85, 0.88)
**Residence** (Rural vs. Urban)	1.20 (1.14, 1.27)	1.66 (1.62, 1.71)	0.57 (0.54, 0.60)	1.16 (1.15, 1.18)
**Comorbidities** (Ref: 0)				
1	1.12 (1.06, 1.17)	1.09 (1.06, 1.12)	1.12 (1.08, 1.17)	1.05 91.04, 1.06)
2	1.21 (1.14, 1.28)	1.10 (1.06, 1.14)	1.13 (1.08, 1.18)	1.03 (1.01, 1.04)
3+	1.39 (1.31, 1.47)	1.19 (1.15, 1.23)	1.19 (1.14, 1.24)	1.05 (1.03, 1.06)
**Anatomical surgical site** (Ref: Knee)				
Hip	0.85 90.81, 0.89)	0.94 (0.91, 0.96)	1.17 (1.13, 1.20)	1.32 (1.31, 1.34)
Spine	0.98 (0.91, 1.06)	1.00 (0.95, 1.05)	0.78 (0.73, 0.840	0.59 (0.57, 0.61)
Elbow/Shoulder	0.89 (0.79, 1.01)	0.83 (0.77, 0.90)		0.61 (0.58, 0.64)
Hand/Wrist	1.22 (1.06, 1.40)	1.16 (1.07, 1.26)	0.12 (0.08, 0.17)	0.14 (0.12, 0.16)
Foot/Ankle	0.86 (0.74, 1.01)	0.94 (0.86, 1.03)		0.36 (0.33, 0.40)
**Extended LOS**[§] (Yes vs. No)	1.07 (1.03, 1.12)	0.98 (0.95, 1.00)	1.57 (1.52, 1.62)	1.39 (1.38, 1.41)
**Adverse events** (1+ vs. 0)	1.36 (1.26, 1.47)	1.17 (1.11, 1.22)	1.42 (1.35, 1.49)	1.04 (1.02, 1.06)

[§] Rate ratios obtained from regression model with log link function and Poisson distribution using Generalized Estimating Equations to account for clustering within hospitals. Surgical sites were grouped as knee, hip spine and other (Elbow/shoulder, hand/wrist, foot/ankle) in the models for inpatient rehabilitation to accommodate smaller sample sizes.

Table S4. Peri-operative and 90-day post-operative outcomes among patients undergoing surgery for osteoarthritis by gradients of pre-operative healthcare use and surgical anatomical site. Ontario, Canada, 2015/16-2017/18.

	**Pre-operative healthcare use gradients n (%)**			
	**Low users** (<50th percentile)	**Moderate users** (50th – 89th percentile)	**High users** (90th – 94th percentile)	**Very high users** (≥95th percentile)
**Knee**				
n	32343	33978	3982	3738
**Peri-operative outcomes**				
Extended LOS	8991 (27.8)	12737 (37.5)	1735 (43.6)	1653 (44.2)
Complications	946 (2.9)	1497 (4.4)	224 (5.6)	228 (6.1)
**Post-operative outcomes**				
Readmission rates	2087 (6.5)	2787 (8.2)	428 (10.7)	539 (14.4)
ED visits	5348 (16.5)	7005 (20.6)	1068 (26.8)	1189 (31.8)
Inpatient rehabilitation	2598 (8.0)	4810 (14.2)	747 (18.8)	823 (22.0)
Home care use	15648 (48.4)	18270 (53.8)	2302 (57.8)	2170 (58.1)
**Hip**				
n	16885	15057	1647	1471
**Peri-operative outcomes**				
Extended LOS	5991 (35.5)	7198 (47.8)	853 (51.8)	799 (54.3)
Complications	774 (4.6)	1017 (6.8)	137 (8.3)	141 (9.6)
**Post-operative outcomes**				
Readmission rates	882 (5.2)	1161 (7.7)	168 (10.2)	177 (12.0)
ED visits	2618 (15.5)	2930 (19.5)	423 (25.7)	457 (31.1)
Inpatient rehabilitation	1644 (9.7)	2706 (18.0)	407 (24.7)	434 (29.5)
Home care use	11455 (67.8)	11007 (73.1)	1255 (76.2)	1129 (76.8)
**Spine**				
n	2253	3687	574	656
**Peri-operative outcomes**				
Extended LOS	764 (33.9)	1560 (42.3)	277 (48.3)	328 (50.0)
Complications	198 (8.8)	378 (10.3)	65 (11.3)	84 (12.8)
**Post-operative outcomes**				
Readmission rates	124 (5.5)	339 (9.2)	67 (11.7)	94 (14.3)
ED visits	371 (16.5)	793 (21.5)	162 (28.2)	188 (28.7)
Inpatient rehabilitation	135 (6.0)	356 (9.7)	84 (14.6)	118 (18.0)
Home care use	523 (23.2)	1142 (31.0)	235 (40.9)	291 (44.4)

**Shoulder & Elbow**				
n	1645	1587	180	183
**Peri-operative outcomes**				
Extended LOS	47 (2.9)	133 (8.4)	25 (13.9)	35 (19.1)
Complications	30 (1.8)	42 (2.6)	8 (4.4)	8 (4.4)
**Post-operative outcomes**				
Readmission rates	74 (4.5)	115 (7.2)	20 (11.1)	36 (19.7)
ED visits	216 (13.1)	303 (19.1)	30 (16.7)	44 (24.0)
Inpatient rehabilitation	-	-	-	-
Home care use	287 (17.4)	530 (33.4)	83 (46.1)	91 (49.7)
**Hand & Wrist**				
n	1099	900	94	110
**Peri-operative outcomes**				
Extended LOS	-	-	-	-
Complications	-	-	-	-
**Post-operative outcomes**				
Readmission rates	77 (7.0)	82 (9.1)	12 (12.8)	13 (11.8)
ED visits	197 (17.9)	221 (24.6)	26 (27.7)	36 (32.7)
Inpatient rehabilitation	-	-	-	-
Home care use	39 (3.5)	59 (6.6)	12 (12.8)	17 (15.5)
**Foot & Ankle**				
n	1535	930	107	109
**Peri-operative outcomes**				
Extended LOS	48 (3.1)	59 (6.3)	12 (11.2)	11 (10.1)
Complications	-	-	-	-
**Post-operative outcomes**				
Readmission rates	54 (3.5)	76 (8.2)	10 (9.3)	15 (13.8)
ED visits	216 (14.1)	173 (18.6)	27 (25.2)	40 (36.7)
Inpatient rehabilitation	-	-	-	-
Home care use	145 (9.4)	194 (20.9)	28 (26.2)	33 (30.3)

- Data not releasable due to small sample size
